# Applying an extended Health Policy Analysis framework to digitally mediated health system reform: Evidence from Saudi Arabia’s Health Sector Transformation Program

**DOI:** 10.1371/journal.pone.0350168

**Published:** 2026-06-08

**Authors:** Ahmed Abdullah Alshehri, Asaad Abdulrahman Abduljawad

**Affiliations:** 1 Department of Health Care Management and Health Informatics, College of Health Sciences, Umm Al-Qura University, Mekkah, Saudi Arabia; 2 Department of Public Health, College of Health Sciences, Umm Al-Qura University, Mekkah, Saudi Arabia; Liverpool John Moores University, UNITED KINGDOM OF GREAT BRITAIN AND NORTHERN IRELAND

## Abstract

Health policy analysis frameworks play a central role in understanding how reforms are designed, implemented, and experienced in practice. Walt and Gilson’s Health Policy Analysis Triangle has been widely used to examine the interaction between policy content, context, actors, and processes, particularly in settings where implementation is shaped by political and organizational dynamics. However, many contemporary health reforms are increasingly digitally mediated and culturally embedded, raising questions about whether existing frameworks sufficiently capture the conditions that structure implementation. This paper proposes a conceptual extension of the Health Policy Analysis Triangle by theorizing Technology and Culture as cross-cutting dimensions that shape how policy content is enacted through actors and processes within specific contexts. The extension is grounded in secondary analysis and theoretical interpretation of empirical patterns previously identified in an evaluation of Saudi Arabia’s Health Sector Transformation Program, a large-scale reform initiative implemented under Vision 2030. Drawing on previously collected policy documents and qualitative interview data from the doctoral study [6] SHSTP evaluation, the paper illustrates how digital infrastructure and sociocultural norms operate as structuring influences on coordination, accountability, participation, and patient-centred care. The proposed framework does not replace the original triangle but enhances its analytical adequacy for reforms unfolding in digitally mediated and culturally complex systems. By making Technology and Culture explicit, the extended model provides a pragmatic analytical framework for analyzing implementation variation and reform learning in Saudi Arabia, with potential relevance for other health systems undergoing rapid transformation when adapted to local contexts.

## 1. Introduction

Analytical frameworks are not simply descriptive tools; they shape what can be seen, compared, and explained in studies of health system reform. In complex reform settings, where priorities shift over time and implementation is negotiated across multiple levels, frameworks help clarify the relationship between policy intent and practice. One of the most widely used approaches in health policy analysis is Walt and Gilson’s Health Policy Analysis Triangle, which foregrounds the interaction between policy content, context, process, and actors [1,2]. Its enduring relevance lies in its capacity to move analysis beyond formal policy texts, drawing attention to the political, organizational, and professional realities that shape policy enactment.

Yet the conditions under which contemporary health reforms unfold have changed in ways that place pressure on established models. In reforms where digitization is central, technology shapes governance and power, not merely implementation tools. Digital technologies now sit at the center of governance and service delivery, altering how decisions are made, monitored, and evaluated. Electronic medical records, telemedicine platforms, digitally mediated referral pathways, and performance dashboards are not merely implementation tools; they can reconfigure accountability, redistribute power between actors, and generate new forms of compliance and resistance. At the same time, sociocultural dynamics continue to influence how reforms are interpreted and experienced. Family involvement in decision-making, religious norms and practices, gendered expectations, professional hierarchies, and workforce diversity can shape the uptake of patient-centered reforms and the practical meaning of “engagement” and “shared decision-making” in everyday care. For these reasons, reform analysis increasingly requires conceptual approaches that attend to the interaction between policy design and the technological and cultural infrastructures through which policy is operationalized [3].

Saudi Arabia’s Health Sector Transformation Program (SHSTP), introduced as part of Vision 2030, offers a particularly instructive setting in which to examine these tensions. The SHSTP has sought to modernize healthcare delivery through structural reforms, including the creation of hospital clusters and the strengthening of care coordination, alongside an explicit emphasis on patient-centered care and chronic disease management. Digital health has been positioned as a core enabling strategy within this reform agenda, with reforms relying on strengthened information systems, technology-enabled continuity of care, and new governance mechanisms designed to support performance-based implementation. Such ambitions are consistent with international policy directions that frame digital infrastructure as a prerequisite for system learning, coordinated care, and reform sustainability [3]. Key initiatives under the SHSTP digital transformation agenda include the expansion of electronic health records, telemedicine services, integrated referral systems, and national digital platforms designed to support coordinated care, performance monitoring, and data-driven governance across hospital clusters. From a theoretical perspective, digitally mediated reforms reshape health system governance by influencing information flows, accountability structures, and coordination mechanisms across healthcare organizations. Digital infrastructures such as electronic health records, telemedicine platforms, and data dashboards therefore function not only as technical tools but also as institutional mechanisms that structure how policy goals are operationalized and monitored. Analytical frameworks that incorporate these dynamics are therefore important for understanding how contemporary health system reforms unfold in practice.

However, experience across health systems also suggests that digital transformation is rarely uniform: its benefits depend on organizational readiness, workforce capability, and the alignment between technology, clinical practice, and governance structures.

Walt and Gilson’s triangle [1] remains a compelling starting point for examining reforms such as the SHSTP because it directs attention to the practical realities of implementation: who drives change, how policy is interpreted, what constraints shape delivery, and where gaps between intent and outcome emerge. Nevertheless, empirical work on digitally enabled reforms suggests that technology and culture frequently operate as more than contextual background. They can structure the actor landscape, reshape implementation processes, and influence how policy content is translated into practice. In the Saudi setting, for example, digital adoption and workforce readiness may condition whether patient-centered care is operationalized through coordinated information flows, while cultural norms may shape communication patterns, family participation, and expectations about authority and decision-making. These dynamics are visible within each of the triangle’s original domains, but the framework does not explicitly conceptualize Technology and Culture as interacting dimensions that can systematically alter policy processes and outcomes.

This paper therefore applies and extends the Health Policy Analysis Triangle to strengthen its analytical usefulness for examining health system reforms that are digitally mediated and culturally embedded. Drawing on empirical insights generated through an evaluation of Saudi Arabia’s Health Sector Transformation Program (SHSTP), the analysis makes Technology and Culture explicit as cross-cutting dimensions that shape how policy content is operationalized, how actors exercise agency, and how implementation processes unfold in practice.

Rather than proposing a purely theoretical reformulation, the paper illustrates how this extended framework can be used as an applied analytical tool to explain variation in reform implementation across settings with differing levels of digital readiness and sociocultural organization. The aim is not to replace the classic triangle, but to enhance its explanatory capacity for contemporary reforms by clarifying how digital infrastructures and cultural norms function as structuring conditions of implementation.

By situating this framework within the empirical context of SHSTP, the paper offers a pragmatic approach for analyzing digitally enabled health system reforms in Saudi Arabia, with potential relevance for other low- and middle-income countries when adapted to local contexts.

## 2. Existing Frameworks and Gaps

### 2.1. The Health Policy Analysis Triangle and its continuing relevance

**Fig 1:**
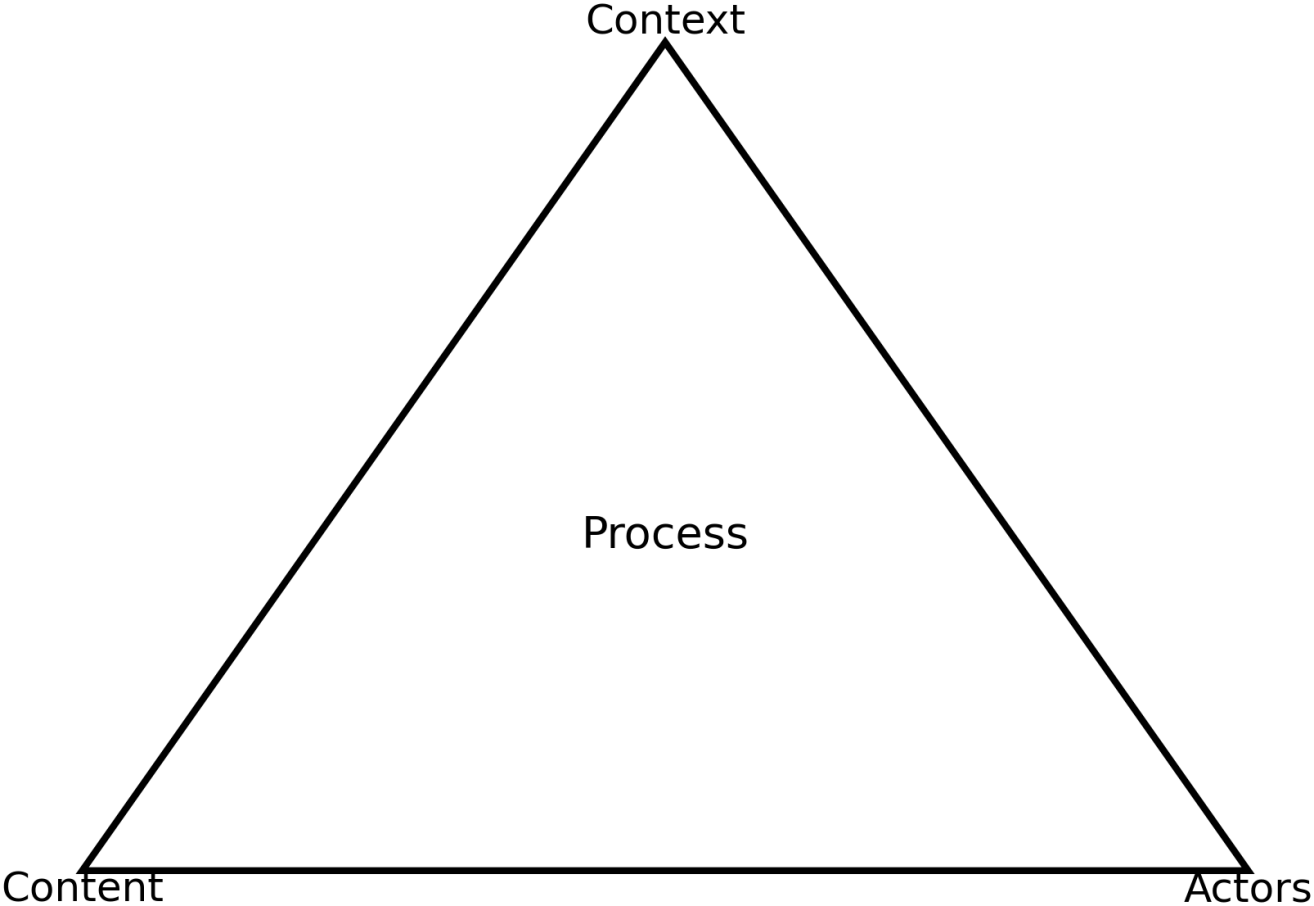
Walt and Gilson’s Health Policy Analysis Triangle (1994).

Analytical frameworks remain central to health policy and systems research because they offer structured ways to examine how reforms are formulated, implemented, and experienced. Walt and Gilson’s Health Policy Analysis Triangle has been particularly influential in this regard, providing a concise heuristic for analysing the interaction between policy content, context, process, and actors. The model helped to shift attention away from policy texts alone toward the political, organizational, and professional dynamics that frequently determine whether reforms are enacted as intended [4]. Its parsimony and portability have contributed to its widespread use across diverse health system reforms, especially in settings where policy outcomes cannot be understood without attending to implementation realities and power relations among stakeholders. The classic framework used in the study is illustrated in **Fig 1**.

The classic policy triangle illustrates the core analytical domains of content, context, actors, and process used to examine health policy formulation and implementation. In mixed-methods evaluation of the Saudi Health Sector Transformation Program (SHSTP), the triangle provides an organizing dimension through which reforms are interpreted and compared across clustered and non-clustered hospitals. The author’s doctoral study [5] illustrates the value of the framework for linking national policy ambitions to service delivery conditions by tracing how reform content (e.g., clustering arrangements and digital health strategies) is shaped by context (e.g., infrastructure readiness and workforce composition), mediated through implementation processes (e.g., phased roll-out and performance governance), and negotiated by actors at different levels of the system.

### 2.2. Analytical limits in digitally mediated health reforms

While the triangle remains analytically useful, contemporary reforms increasingly unfold through digitally mediated systems of governance and care delivery. Digital infrastructures, such as electronic medical records, telemedicine platforms, referral and triage systems, and performance dashboards, operate as more than implementation supports. They shape what is measurable, how accountability is organized, and how decision-making authority is distributed across actors. WHO’s global strategy on digital health frames digital transformation as a governance and systems issue that depends on institutional readiness, workforce capability, and sustainable coordination rather than technology acquisition alone [6]. OECD analyses similarly emphasize that digital health adoption is inseparable from questions of policy capacity, equity, interoperability, and data governance [7].

These concerns resonate strongly with the SHSTP implementation experience documented in the author’s doctoral study [5]. Differences between clustered and non-clustered hospitals are repeatedly explained through digital readiness and integration into clinical workflows: clustered hospitals report stronger coordination facilitated by information systems and virtual care tools, whereas non-clustered hospitals face constraints linked to uneven infrastructure, inconsistent use, and limited training [5]. Such findings align with broader implementation evidence showing that health information technology improves performance only when embedded into organizational routines and supported by governance arrangements that align digital systems with clinical and managerial practice [8]. They also reflect sociotechnical insights that digital innovations frequently generate new forms of work, resistance, and adaptation, which can shape policy implementation trajectories in ways not captured when technology is treated as a sub-element of context [9]. Analysis of the SHSTP implementation indicates that technology influences reform processes through a small number of recurring and analytically identifiable mechanisms.

### Mechanisms: how Technology reshapes implementation

The mechanisms presented here were derived through iterative analysis of recurring patterns identified across interview and documentary data. These mechanisms were selected because they consistently appeared across multiple data sources and demonstrated clear influence on implementation processes, rather than representing isolated or context-specific observations. The original policy triangle can incorporate technology indirectly, typically under content or context, but it does not explicitly conceptualize digital infrastructure and data-driven governance as dimensions that can actively reshape actor relations, implementation processes, and reform outcomes. In reforms where digitization is foundational to policy design, this analytic under-specification becomes increasingly consequential.

In the extended framework, Technology reshapes implementation through four mechanisms

Interoperability: Enables or constrains continuity of care and referral pathways regardless of stated policy intent, shaping whether integrated service delivery is operationally feasible.

Measurement regimes: Define what is visible, auditable, and comparable, thereby shaping organizational priorities, professional behavior, and interpretations of performance.

Digital governance: Redistributes accountability and authority toward actors who control data standards, reporting requirements, and digital platforms, influencing decision-making power.

Workflow embedding and capability: Determines whether digital tools are adopted, adapted, or bypassed in practice, shaping implementation trajectories through routinisation, workarounds, or non-adoption.

### 2.3. Cultural dynamics and the limits of treating culture as “background context”

A second conceptual gap concerns the handling of sociocultural dynamics. In many applications of the triangle, culture is absorbed into “context,” which risks treating it as a passive background condition rather than a force that shapes how engagement, authority, trust, and decision-making are enacted at the point of care. This is particularly relevant in reforms that rely on strengthening patient-centered care and shared decision-making, where the meaning of participation and autonomy varies across cultural and institutional settings.

In the Saudi context, the author’s doctoral study [5] shows that family involvement, religious norms, gendered interaction expectations, and professional hierarchies influence how patient-centered care is operationalized and experienced across hospitals. For example, family participation in care decisions can function as a facilitator of adherence and support, yet it may also mediate how “patient voice” is expressed in clinical encounters. Similarly, culturally sensitive communication practices can shape trust and satisfaction, particularly within chronic disease management where continuity and engagement are central. These dynamics interact with policy actors and implementation processes, influencing whether reforms are enacted as intended rather than simply shaping the environment in which implementation occurs. Empirical findings from the SHSTP evaluation further suggest that sociocultural dynamics influence implementation through several recurring mechanisms that operate across actors and processes.

### Mechanisms: how Culture reshapes implementation

The mechanisms presented here were derived through iterative analysis of recurring patterns identified across interview and documentary data. These mechanisms were selected because they consistently appeared across multiple data sources and demonstrated clear influence on implementation processes, rather than representing isolated or context-specific observations. While culture is often subsumed under “context” in applications of the policy triangle, evidence from SHSTP implementation indicates that sociocultural dynamics actively reshape how policy intent is interpreted, enacted, and experienced. In the extended framework, Culture reshapes implementation through four mechanisms:

Family-mediated decision-making: Alters the meaning of “patient voice” and shared decision-making by embedding clinical decisions within family authority structures.

Religious norms: Shape acceptability, adherence, and care routines, influencing when and how services are accessed and delivered (for example, during Ramadan).

Gender norms: Condition access, comfort, and communication patterns between patients and clinicians, affecting engagement and information exchange.

Hierarchy and authority norms: Shape how questions are asked, consent is negotiated, and trust is formed, influencing the distribution of agency among actors.

Religious norms were treated as a distinct mechanism because they shape acceptability, care timing, and adherence through normative frameworks that operate independently of professional hierarchy (for example, fasting practices or gender-sensitive care expectations). In contrast, hierarchy and authority norms structure interpersonal communication and decision-making within clinical encounters, particularly in relation to professional roles and expectations of deference.

### 2.4. The gap this paper addresses

Taken together, the SHSTP evaluation suggests that the classic triangle remains a strong starting point for analyzing reform, but it is under-specified for settings where digitization and sociocultural dynamics function as structuring conditions of implementation.

When technology and culture are treated only as background context, policy analysis risks remaining descriptive rather than explanatory. Such treatment obscures how digital infrastructures actively restructure accountability, coordination, and discretion, and how sociocultural norms shape participation, legitimacy, and decision-making at the point of implementation. As a result, variation in reform outcomes may be attributed vaguely to “context” rather than examined through identifiable mechanisms linking policy content, actors, and implementation processes.

This analysis therefore points toward the need for a refined analytical framework that preserves the strengths of Walt and Gilson’s original triangle while addressing its limitations in digitally mediated and culturally embedded reform settings. Evidence from the Saudi Health Sector Transformation Program indicates that digital infrastructure and sociocultural conditions operate as structuring influences that shape policy content, actor capacity, implementation processes, and contextual constraints in systematic ways. While these influences are visible across the triangle’s domains, they are not explicitly conceptualized within the classic framework. This paper responds to that gap by applying and extending the policy triangle that retains its analytic core while making Technology and Culture explicit as interacting dimensions, grounded in the doctoral evaluation of SHSTP implementation. The study explicitly recognizes the limitation of the classic triangle, noting that it can imply linearity and offers limited conceptual space for temporal evolution, feedback mechanisms, and emergent behaviors that characterize long-term reforms.

The author’s doctoral study [5] also highlights that in highly centralized systems, structural reforms such as clustering and technology-enabled governance tools can become mediators of reform outcomes, while “context” may need to be expanded to include organizational maturity, infrastructure, and frontline readiness.

This paper therefore argues for an extension of Walt and Gilson’s framework that makes Technology and Culture explicit interacting dimensions alongside content, context, process, and actors. The purpose is not to replace the original model, but to strengthen its analytical adequacy for digitally mediated and culturally embedded reforms such as SHSTP under Vision 2030. By conceptualizing technology and culture as dimensions that can systematically reshape relationships among actors, implementation processes, and policy effects, the proposed extension offers a pragmatic and locally contextualized framework that may have relevance for other low- and middle-income settings pursuing health system transformation, provided it is adapted to local institutional, technological, and sociocultural contexts.

## 3. Conceptual methodology

This paper develops an applied analytical extension of Walt and Gilson’s Health Policy Analysis Triangle through an inductive, interpretive process grounded in empirical material generated during a mixed-methods evaluation of the Saudi Health Sector Transformation Program (SHSTP). The original study used the policy triangle as the primary analytic dimension to examine implementation and outcomes across clustered and non-clustered hospital settings. For the purposes of this article, no new data are analyzed. Instead, the extension is derived from a structured re-examination of the study’s qualitative and documentary evidence, with attention to recurring implementation mechanisms related to digitization and sociocultural conditions that were not fully articulated within the classic framework.

### 3.1. Empirical foundations for concept development

Two empirical sources informed this conceptual adaptation. First, the study included a structured analysis of policy and organizational documents capturing the reform architecture and governance arrangements under SHSTP, including strategic and operational materials, accountability tools, and guidance for technology-enabled service delivery. Second, the study drew on semi structured interviews with patients and senior physicians, producing accounts of how reforms were enacted and experienced in practice, including perceived changes in patient-centered care, constraints on engagement, and the operational implications of digital health adoption. The interview approach combined consistent prompts with open-ended probing, enabling both comparability across participants and the emergence of unanticipated themes relevant to conceptual development. The qualitative interview dataset used in this study was originally collected as part of a doctoral evaluation of the Saudi Health Sector Transformation Program. The present paper does not introduce new empirical data but develops a conceptual extension of the Health Policy Analysis Triangle through secondary analytical interpretation of the existing dataset. The doctoral thesis [5] from which the dataset originates provides the full empirical analysis on which the present conceptual interpretation builds. This study should therefore be understood as a conceptual and interpretive extension grounded in secondary analysis of an existing qualitative dataset, rather than as a primary empirical investigation.

### 3.2. Analytic reasoning: from patterns to conceptual abstraction

The analytic process followed a structured, step-by-step approach to move from empirical observations to conceptual abstraction, ensuring transparency in how patterns were identified and interpreted. This pattern-to-abstraction logic is commonly used in qualitative policy analysis to move from empirical observations to structured analytical categories. The analytic task was not to generate a new theory de novo, but to extend an established framework by identifying recurrent empirical pressures that were analytically consequential yet under-specified in the parent model. Four steps summarize the reasoning process.

#### Step 1: Triangulated mapping of reform intent and implementation experience.

Documentary evidence was examined alongside stakeholder narratives to identify where reform intent aligned with operational reality, and where patterned discrepancies emerged. This triangulated reading reduced over-reliance on official policy representations or individual accounts and strengthened interpretive credibility. This step involved systematic comparison of documentary and interview data to identify areas of convergence and divergence in implementation experiences.

#### Step 2: Coding anchored in the original triangle, with deliberate space for emergence.

The study analysis was structured around the classic domains (content, context, process, actors) to maintain theoretical coherence, while inductive coding was used to capture themes that extended beyond the explanatory reach of the original framework. This combined deductive–inductive approach was central to conceptual adaptation. Codes were generated iteratively and refined through repeated reading of the data, with attention to both predefined analytical categories and emergent themes. It preserves continuity with the parent model while allowing empirically grounded extensions to be articulated.

#### Step 3: Identification of cross-cutting pressures insufficiently explained by the classic categories.

Across the SHSTP dataset, two cross-cutting pressures repeatedly shaped implementation trajectories: (a) technology-enabled governance and digital infrastructure, and (b) sociocultural conditions shaping engagement, legitimacy, and decision-making. These were identified based on their recurrence across multiple interviews and documentary sources and their consistent influence on implementation processes. These were not peripheral contextual features. They functioned as mechanisms that reconfigured actor capacity, mediated implementation pathways, and influenced how patient-centered objectives were operationalized across settings.

These cross-cutting pressures were identified through iterative comparison of coded data, where themes that appeared consistently across multiple interviews and were corroborated by documentary sources were retained as analytically significant mechanisms shaping implementation processes.

#### Step 4: Conceptual consolidation into explicit extensions of the framework.

The final step translated these recurrent pressures into explicit analytical extensions, **Technology** and **Culture**, and specified their interaction with the original domains rather than treating them as subsidiary elements absorbed within “context.” This consolidation was guided by the study’s theoretical discussion, which emphasizes that in centralized reform settings, structural arrangements and technology-enabled governance tools can mediate outcomes, and that operational context includes organizational maturity, infrastructure readiness, and frontline capability. The study also supports greater granularity in the “actors” domain by distinguishing strategic and operational actors with different capacities to shape implementation.

### 3.3. Coherence and trustworthiness

The study incorporated credibility checks through participant engagement, with confirmatory input indicating that the interpreted patterns resonated with respondent experience. For this conceptual paper, coherence was strengthened by ensuring that each proposed modification could be traced to consistent empirical patterns across documentary and interview evidence, and by maintaining the integrity of the original triangle as the analytic core rather than replacing it. No additional data collection or re-analysis was undertaken for this paper; all analytical extensions are grounded in patterns identified within the existing SHSTP evaluation dataset. The empirical insights from the SHSTP evaluation and their analytical implications are summarized in Table 1.

**Table 1:** Mapping SHSTP empirical insights to proposed analytical extensions of the policy triangle.

SHSTP empirical insight	Analytic implication for the classic triangle	Proposed conceptual modification
Documentary evidence shows digitization embedded in the reform architecture through governance instruments, implementation guidance, and accountability tools.	Technology is not only a tool used during implementation; it is built into how reform priorities are operationalized and monitored.	Add a **Technology** dimension to analyze digital governance, infrastructure readiness, interoperability, and data-driven accountability.
Interview data indicate patterned differences between clustered and non-clustered settings that are partly explained by digital readiness, workflow integration, and capability.	“Context” and “process” alone do not fully capture how digital capacity conditions what implementation is feasible.	Treat **Technology** as an interacting dimension shaping actor capacity, implementation pathways, and practical outcomes.
Analysis was anchored in triangle domains but required inductive categories to capture recurring themes extending beyond the classic model.	The triangle provides coherence, but empirical material generates additional constructs that recur across domains.	Justify the extension as a **conceptual adaptation**: retain the core and add dimensions grounded in recurrent themes.
Sociocultural dynamics influence how patient-centered reforms are enacted (e.g., family participation in decisions, religious and gendered norms shaping engagement).	Culture is not merely background context; it shapes legitimacy, communication, engagement, and decision-making at the point of care.	Add a **Culture** dimension to examine norms, family-mediated decision-making, authority relations, and culturally shaped expectations.
Reform governance indicates that structural arrangements and technology-enabled tools can mediate reform effects in centralized systems.	The triangle under-specifies mediating mechanisms through which structures and tools shape implementation.	Specify **mediating mechanisms** (technology-enabled governance and structural arrangements) that condition how content is enacted through process and actors.
Evidence highlights the importance of organizational maturity, infrastructure, and frontline readiness in determining implementation success.	Operational context requires explicit attention to meso- and micro-level capability, not only macro sociopolitical conditions.	Expand the operationalization of **Context** and show its interaction with Technology and Culture.
Actor influence differs by level (strategic actors vs operational actors), with uneven agency in shaping implementation.	The actor category benefits from greater granularity to reflect unequal capacity and decision authority.	Introduce **actor stratification** (strategic vs operational) within the revised framework.
The study notes the challenge of analyzing long-term reforms using static representations that underplay feedback, evolution, and emergent change.	A static triangle can understate dynamic implementation cycles and learning loops.	Explicitly incorporate **interaction and feedback** across domains, especially where digital dashboards and cultural legitimacy shape iterative reform responses.

### 3.4. Qualitative analytical procedures

Interview transcripts and policy documents were analyzed using a qualitative thematic approach. The analysis combined deductive coding based on the Health Policy Analysis Triangle with inductive coding to capture emerging themes related to digital governance and sociocultural dynamics. Coding and theme development were conducted using NVivo software.

Coding was conducted by the primary researcher using an iterative approach, with repeated review of transcripts and documentary data to ensure consistency in code application and theme development. Given the interpretive nature of the study, emphasis was placed on analytical coherence and transparency rather than formal intercoder reliability measures.

Patterns were compared across interviews and documentary sources to identify recurring implementation mechanisms. The analytical process focused on linking empirical observations to the conceptual refinement of the framework. Recurring mechanisms related to technology-enabled governance and sociocultural dynamics were identified through iterative coding and comparison across interview transcripts and documentary evidence. The analytical process followed an iterative interpretive approach in which emerging themes were repeatedly compared with documentary evidence and interview accounts to ensure conceptual coherence and transparency.

### 3.5. Ethical consideration

The study underpinning this conceptual analysis was conducted in accordance with the Declaration of Helsinki and received ethical approval from the Research Ethics Committee of the College of Health Sciences at Swansea University (protocol code 280818; approved 12 July 2019) and from Umm Al-Qura University, Saudi Arabia (protocol code 2019/001; approved 27 August 2019). Written informed consent was obtained from all participants prior to data collection. All interview and documentary data were de-identified before analysis to protect participant confidentiality.

No additional data collection or re-analysis was undertaken for the present paper. The conceptual extensions proposed here are based on secondary analysis and theoretical interpretation of patterns identified within the existing Saudi Health Sector Transformation Program (SHSTP) evaluation dataset. Access to any de-identified subset of the interview data is subject to approval by the relevant institutional ethics and governance procedures because the study involves confidential human participant materials.

## 4. Extended model presentation

**Fig 2:**
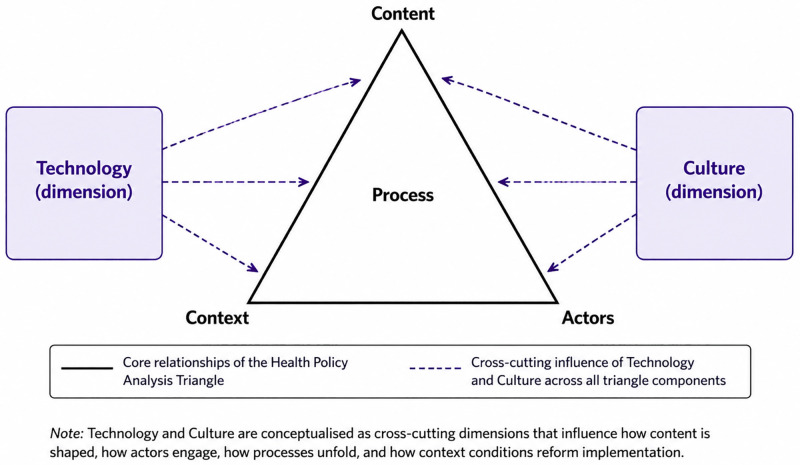
Extended Policy Triangle: Technology and Culture as cross-cutting dimensions.

This paper extends Walt and Gilson’s triangle by conceptualizing Technology and Culture as cross-cutting dimensions shaping SHSTP implementation. This section presents an applied analytical extension of Walt and Gilson’s Health Policy Analysis Triangle for examining digitally mediated and culturally embedded reforms. The argument is not that the triangle is obsolete. On the contrary, its enduring value lies in its ability to organize policy analysis around the interaction between content, context, actors, and process [1,2]. The limitation is that many contemporary reforms are enacted through infrastructures and social relations that are not adequately captured when technology and culture are treated only as background context. Evidence from SHSTP indicates that digital systems and sociocultural norms function as structuring conditions that shape what implementation is feasible, how reform is governed and measured, and how patient-centered objectives are interpreted in everyday practice. The extended model therefore retains the triangle as the analytic core while making Technology and Culture explicit as cross-cutting analytical dimensions. The proposed conceptual extension of the framework is presented in **Fig 2**.

### 4.1. Retaining the analytic core: content, context, actors, and process

The extended framework preserves the conceptual logic of the original triangle. Content refers to the substance of the reform: the goals, policy instruments, standards, pathways, incentives, and performance expectations. Context refers to the conditions within which reform is enacted, including organizational capacity, infrastructure, workforce composition, institutional arrangements, and broader political and economic constraints. Actors include stakeholders with formal or informal influence over policy design and implementation, such as ministries, regulators, managers, clinicians, patients, and families. Process refers to the practical work through which policy is translated into action, including planning and roll-out, coordination routines, monitoring systems, feedback mechanisms, and iterative revision.

This core remains essential for analyzing SHSTP because the reform involves multiple interacting policy instruments operating across levels of the system, and outcomes are shaped by how actors interpret and negotiate implementation within specific contexts over time. What the extension adds is explicit analytical space to examine how digital infrastructures and sociocultural norms restructure the operation of these domains and the relationships among them.

### 4.2. Technology as a structuring dimension of implementation

In the extended framework, Technology is treated as a distinct dimension because digital infrastructures increasingly shape implementation pathways in ways that cannot be fully captured when technology is treated only as a sub-element of context or as a policy instrument within content. Technology is conceptualized here as the constellation of digital tools, information infrastructures, governance arrangements, and capabilities through which reform is operationalized and monitored. Four elements are particularly salient.

First, integration and interoperability. Digital systems determine whether information can circulate across units, facilities, and levels of care, thereby enabling or constraining continuity, coordination, and referral management. Where interoperability is limited, the operational logic of integrated reforms is restricted irrespective of policy intent [8].

Second, data systems and measurement regimes. Digital infrastructures shape what can be recorded, audited, and compared, and therefore influence which forms of work are made visible and valued. In performance-oriented reforms, digitally mediated reporting can elevate measurable activities while rendering relational or interpretive aspects of patient-centered practice less visible. Digital health strategies therefore operate as governance arrangements as much as technical platforms.

Third, digital governance and accountability. Technology enables new modes of monitoring, reporting, and performance management, including real-time dashboards and automated reporting. These mechanisms may improve coordination and responsiveness, but they can also create new compliance burdens and shift decision authority toward those who control data standards, reporting requirements, and platform access. In this way, technology mediates actor relationships and reshapes the implementation process itself.

Fourth, workflow embedding and capability. Evidence from health information technology research shows that benefits are contingent on workflow fit, training, organizational readiness, and sustained support. Systematic reviews demonstrate that health IT can improve quality and efficiency, but effects are uneven and depend on implementation quality and the alignment between technology, governance, and clinical practice [8]. Sociotechnical scholarship similarly emphasises that non-adoption, abandonment, and uneven scale-up are common in complex technology-enabled change [9].

Interaction with the original domains. Technology interacts with each component of the original triangle. It shapes content when policy instruments include digital requirements (platform use, reporting standards, interoperability expectations). It shapes context because infrastructure readiness and digital capability become material constraints on implementation. It shapes actors by creating new roles and asymmetries (for example, those with access to data systems or authority over reporting). It shapes process because implementation becomes partly an ongoing exercise in configuring, stabilizing, and adapting digital systems, with policy learning mediated through data flows and performance feedback.

### 4.3. Culture as an interface shaping legitimacy, engagement, and enactment

In the extended framework, Culture is conceptualized as an interacting dimension because sociocultural conditions can shape implementation through mechanisms that operate across actors, processes, and the practical meaning of reform objectives. In reforms that emphasize patient-centered care, chronic disease management, and engagement, the meaning of participation and the distribution of decision authority cannot be assumed to be culturally neutral. Culture in this framework refers to shared norms and expectations that shape communication, trust, legitimacy, and decision-making in clinical and organizational settings.

Four elements are especially relevant.

First, norms of decision-making and family involvement. In contexts where health decisions are frequently family-mediated, patient-centered care may be enacted through clinician–patient–family interactions rather than through individual autonomy alone. The practical meaning of “shared decision-making” therefore depends on culturally embedded expectations about who participates, whose voice carries authority, and how consensus is reached.

Second, religious and moral frameworks shaping care practices. Religious values can shape understandings of illness, lifestyle guidance, treatment acceptability, and adherence. Such influences affect both the reception of reform aims and the micro-processes through which care is delivered.

Third, gendered and hierarchical interaction norms. Expectations about communication, deference to professional authority, and gender-sensitive interaction can influence whether patients raise concerns, how preferences are voiced, and how engagement is documented. These dynamics have direct implications for whether patient-centered reforms translate into routine practice rather than remaining rhetorical commitments.

Fourth, workforce diversity and professional subcultures. In systems with internationally diverse workforces, linguistic and cultural differences can shape teamwork, communication, patient counselling, and coordination. Such dynamics influence actor relationships and implementation routines, and therefore the policy process.

Interaction with the original domains. Culture interacts with each component of the triangle. It shapes content insofar as policy instruments may assume a particular model of autonomy and engagement that may not align with local decision-making norms. It shapes context through socially patterned expectations and organizational climates that confer legitimacy or generate resistance. It shapes actors because agency and authority are culturally patterned; patients, families, clinicians, and managers operate within normative expectations that influence behavior beyond incentives. It shapes process because implementation depends on culturally mediated practices of communication, counselling, consent, follow-up, and trust-building.

### 4.4. Why Technology and Culture are conceptualized as cross-cutting dimensions

A central design decision in this framework is to represent Technology and Culture as cross-cutting dimensions rather than as additional vertices. This signals that they do not compete with the original domains; they operate through them. Both dimensions shape how content is translated into process, how actors exercise agency, and how contextual constraints are experienced. Making Technology and Culture explicit therefore improves analytical adequacy in two ways. First, it clarifies how identical policy content can produce different outcomes where digital capability and infrastructural integration vary across settings. Second, it captures how culturally embedded norms can shape engagement and legitimacy, affecting whether reforms oriented toward patient-centered care and chronic disease management are enacted in ways that are meaningful to patients and workable for clinicians.

### 4.5. Summary

In summary, the extended framework retains Walt and Gilson’s triangle as the analytic core while strengthening it through explicit recognition of Technology and Culture as cross-cutting dimensions of implementation. This extension is consistent with contemporary digital health policy guidance that conceptualizes digital transformation as a governance and systems-capability issue and with sociotechnical scholarship highlighting the dynamic, adaptive, and uneven nature of technology-enabled change. It offers a pragmatic, locally contextualized policy dimension for examining SHSTP implementation, while also providing a transferable conceptual tool for analyzing digital-era reforms in other low- and middle-income settings.

## 5. Illustrative Vignettes

The following illustrative examples are derived from recurring patterns identified across the interview dataset and documentary evidence collected during the SHSTP evaluation. These vignettes are intended to illustrate typical implementation dynamics observed in the data rather than to represent the frequency or distribution of experiences across the full sample. These examples are presented to demonstrate how the proposed analytical dimensions of Technology and Culture operate within specific implementation contexts.

This section presents three brief mini cases drawn from the SHSTP evaluation to demonstrate how the extended framework operates in practice. Each vignette is structured to make explicit the policy content, the relevant cross-cutting dimension (Technology or Culture), the mechanism through which implementation is reshaped, and the observed effect on reform enactment.

The vignettes are constructed from multiple accounts reflecting similar patterns, rather than from single isolated cases.

### Vignette 1: Technology-enabled follow-up reshapes implementation processes

This vignette reflects patterns observed across approximately 10–15 participants, primarily in clustered hospital settings.

- Policy content: Strengthening continuity of care and follow-up compliance as part of patient-centered chronic disease management under SHSTP.
- Dimension: Technology (telemedicine platforms and digital scheduling tools).
- Mechanism: Workflow embedding of virtual follow-up reduces travel burden and coordination effort, altering service delivery routines for clinicians and patients.
- Observed effect: Follow-up is perceived as easier and more continuous in clustered settings, supporting sustained engagement and adherence.

#### Vignette 2: Digital interoperability conditions coordination across services.

This vignette reflects accounts reported across both clustered and non-clustered hospital settings, indicating variation in implementation linked to differences in system integration.

- Policy content: Improving coordination and integration across departments and levels of care through shared patient information systems.
- Dimension: Technology (electronic medical records and interoperability infrastructure).
- Mechanism: Data accessibility and system integration shape handover processes and coordination routines between clinical units.
- Observed effect: Where interoperability is functional, patients experience seamless care (“all departments can access my records”); where integration is weak, coordination breaks down, producing uneven implementation despite uniform policy intent.

#### Vignette 3: Cultural norms shape participation in clinical decision-making.

This vignette draws on accounts from multiple participants across settings, highlighting recurring sociocultural patterns shaping decision-making practices.

- Policy content: Promoting patient-centered care and shared decision-making within routine clinical encounters.
- Dimension: Culture (family-mediated decision-making norms).
- Mechanism: Sociocultural expectations redistribute decision authority toward family members, reshaping clinician–patient interaction patterns.
- Observed effect: In some settings, family involvement enhances support and adherence; in others, patient voice is partially displaced, illustrating how cultural norms condition the enactment of patient-centered policy goals.

**Table 2:** Illustrative vignettes mapped to the extended framework dimensions. The table summarizes how policy content, actors, and implementation processes are mediated by Technology and Culture in selected SHSTP examples. Short excerpts from participant interviews are included to illustrate recurring implementation patterns identified in the SHSTP evaluation. Excerpts are illustrative and not intended as exhaustive qualitative analysis. Selected illustrative examples demonstrating how Technology and Culture mediate implementation processes are summarized in Table 2.

Vignette	Short excerpt (illustrative)	Content	Context	Process	Actors	Technology	Culture
1. Technology-enabled follow-up	“Follow-ups are much easier online now.” (P12, patient, clustered hospital)	PCC and continuity commitments implicitly supported	Digital readiness enables access	Follow-up routines shift to virtual pathways	Patient–clinician interaction reconfigured	Tele/virtual tools enable follow-up	Indirect (not primary)
2. Interoperability conditions coordination	“All departments can now access my records.” and “My data doesn’t sync well across departments.” (P08, clinician, clustered hospital)	Reform emphasis on integration/coordination	Infrastructure + integration capacity vary	Coordination succeeds or breaks at handovers	Clinicians/patients affected by access barriers	EMR access + data syncing central	Indirect (not primary)
3. Culture shapes participation in decisions	“We ensure that families are part of the discussion…” and “The doctors usually talk more to my sons than me.” (P21, patient, non-clustered hospital)	Patient-centered care interpreted through local norms	Organizational time/capacity affects engagement	Shared decision-making enacted or undermined	Family, patient, clinician roles negotiated	Not primary	Family-mediated decision-making central

## 6. Discussion

This paper advances an analytically grounded extension of Walt and Gilson’s Health Policy Analysis Triangle to strengthen its explanatory adequacy for reforms that are simultaneously digitally mediated and culturally embedded. The classic triangle remains a powerful organizing heuristic because it directs attention to the interaction between policy content, context, actors, and process, and because it highlights that implementation is fundamentally political and negotiated rather than purely technical. The extension advanced here does not displace that logic. Instead, it makes explicit two dimensions that increasingly structure how reforms are governed and experienced: Technology and Culture. These dimensions are not treated as background descriptors but as cross-cutting dimensions that shape the feasibility of implementation, the distribution of actor agency, the functioning of accountability mechanisms, and the practical meaning of patient-centered objectives. While the empirical observations originate from the SHSTP evaluation dataset, the contribution of this paper lies in the conceptual interpretation of those patterns to extend the Health Policy Analysis Triangle.

### 5.1 Theoretical advancement: from contextual factors to cross-cutting dimensions

A central analytical contribution of the extended model is the move from treating technology and culture as “contextual variables” to conceptualizing them as cross-cutting dimensions that operate across the triangle’s domains. Many health policy analyses acknowledge that “context matters,” yet frequently leave context under-specified or treated as a container for diverse influences. This becomes increasingly problematic in reform programs where digital infrastructures and sociocultural expectations systematically shape implementation pathways. By positioning Technology and Culture as cross-cutting dimensions, the model preserves the triangle’s parsimony while making explicit the mechanisms through which digital platforms, data regimes, family-mediated decision-making, and normative expectations shape actor behavior and implementation routines.

This theoretical move is particularly relevant for digital transformation agendas that rely on data systems and platform-enabled governance. Digital infrastructures can elevate particular metrics and behaviors, redistribute authority through control of reporting standards, and shape what counts as “performance” or “quality” in practice. International policy guidance increasingly treats digital health as a governance and capacity issue, requiring institutional readiness, sustainable coordination, and attention to equity, not merely the acquisition of tools [6]. The extended triangle provides a structured way to examine these dynamics without dissolving analysis into an unbounded list of contextual influences. Instead, technology, cultural norms, organizational context, and actor agency interact dynamically to shape how policy intentions are translated into operational practice. The empirical illustrations from the SHSTP case further indicate that these dimensions rarely operate independently; rather, technology, sociocultural norms, organizational context, and actor agency interact dynamically to shape how policy intentions are translated into operational practice.

### 5.2 Comparison with Kingdon: agenda-setting versus implementation architecture

Kingdon’s Multiple Streams Framework is highly effective for explaining how health reforms gain agenda status through the coupling of problems, policies, and political opportunity [10]. This comparison is intended as a conceptual positioning of the extended framework rather than as an empirical finding derived directly from the SHSTP dataset. Its primary strength lies in analyzing reform emergence and timing. However, for SHSTP-type reforms where strategic direction is already established, the key analytical challenge shifts from agenda entry to implementation architecture. Kingdon’s framework offers limited tools for examining how reforms are operationalized through digital infrastructures, governance routines, and frontline practices. The extended policy triangle complements Kingdon by focusing downstream, enabling analysis of how policy intent is enacted, stabilized, and reshaped through technology-enabled coordination and culturally mediated engagement.

### 5.3 Comparison with Sabatier: coalitions and long-term policy change versus delivery-level mediation

The Advocacy Coalition Framework (ACF) explains policy stability and change through competing belief systems, coalition dynamics, and policy-oriented learning over time [11]. This comparison is intended as a conceptual positioning of the extended framework rather than as an empirical finding derived directly from the SHSTP dataset. It is particularly valuable for contested policy domains characterized by ideological conflict. In contrast, SHSTP implementation variation is less driven by coalition competition than by organizational capacity, digital readiness, and governance instruments. The extended triangle addresses this different analytic level by focusing on how implementation is mediated through technology-enabled governance and sociocultural norms, while remaining compatible with ACF for analyzing longer-term political dynamics.

### 5.4 Comparison with Lipsky: discretion, street-level practice, and technology-enabled accountability

Lipsky’s Street-Level Bureaucracy highlights how frontline discretion shapes policy enactment under conditions of constraint and ambiguity [12]. Its relevance to health reform remains strong. However, contemporary reforms increasingly operate through digital systems that restructure discretion itself by embedding rules, metrics, and accountability mechanisms into workflows. The extended triangle builds on Lipsky by specifying Technology and Culture as dimensions through which discretion, compliance, and agency are jointly produced in digitally mediated and culturally patterned implementation contexts.

### 5.5 Linking system reform and digital transformation: why the extension matters

Evidence from the SHSTP evaluation suggests that variation in implementation across clustered and non-clustered hospitals is repeatedly explained through differences in digital capability, system integration, and culturally mediated engagement rather than through policy design alone. Without explicit Technology and Culture dimensions, implementation analysis risks misclassifying key mechanisms as undifferentiated “context,” obscuring how digital infrastructures restructure accountability and how sociocultural norms shape participation and authority. By making these dimensions explicit, the extended triangle enables more precise explanation of implementation variation, particularly between clustered and non-clustered settings under SHSTP. The framework therefore shifts analysis from descriptive contextualization toward mechanism-based explanation of how policy content is enacted through actors and processes.

A practical contribution of the model is that it bridges two bodies of scholarship that are often treated separately: health system reform analysis and digital transformation analysis. Studies of health IT consistently suggest that benefits depend on implementation quality, workflow fit, training, and organizational readiness rather than the mere presence of technology [8]. Sociotechnical work similarly highlights that non-adoption, abandonment, uneven scale-up, and sustainability challenges are common in technology-supported change, especially in complex service settings [9].At the same time, patient-centered reforms depend on legitimacy and engagement, which are often culturally mediated. Without explicit conceptual space for technology and culture, analyses risk describing these forces as peripheral context rather than examining how they restructure actors, processes, and outcomes.

Representing Technology and Culture as cross-cutting dimensions therefore clarifies how identical policy content can yield uneven effects across settings. Where digital systems are integrated and usable, coordination routines and follow-up processes may stabilize and improve. Where integration is weak or capability is limited, reforms may generate new burdens and coordination failures. Similarly, where culturally aligned communication and decision-making routines are established, patient-centered intent may be realized as meaningful engagement; where norms are misaligned or authority relations restrict voice, “patient-centeredness” may be experienced as partial or mediated through family authority. The extended framework provides an organized way to examine these dynamics while preserving the explanatory strengths of the original triangle.

### 5.6 Generalizability and analytic use beyond the Saudi case

Although developed in relation to SHSTP, the extended model is not intended as a context-specific framework applicable only to Saudi Arabia. Rather, it is a pragmatic conceptual refinement designed for settings where reforms rely on digital infrastructures and where implementation is shaped by sociocultural norms of engagement, authority, and legitimacy. These conditions are increasingly common in Gulf health systems and in many low- and middle-income countries pursuing transformation alongside rapid digitization. The model may therefore be useful as an analytic dimension beyond the Saudi case, but its application in other settings should be treated as context-sensitive rather than directly generalizable. Researchers should operationalize Technology and Culture in locally appropriate ways, for example, by specifying digital maturity, interoperability, and data governance on the technology side, and decision-making norms, family roles, communication expectations, and trust patterns on the cultural side.

In summary, the extended policy triangle advances health policy analysis in three ways. First, it preserves the clarity and usability of the classic triangle while improving its fit for digitally mediated reforms. Second, it strengthens implementation analysis by treating Technology and Culture as cross-cutting dimensions that shape content, context, actor agency, and process routines. Third, it offers an analytical coherent bridge between system reform evaluation and digital transformation scholarship, supporting more analytically adequate explanations of implementation variation and reform effects.

## 7. Implications and Conclusion

This paper advances a contemporary update of Walt and Gilson’s Health Policy Analysis Triangle by retaining its analytic core—content, context, actors, and process—while making Technology and Culture explicit as cross-cutting dimensions that shape how reforms are designed, governed, implemented, and experienced [13,4,14].

**Fig 3:**
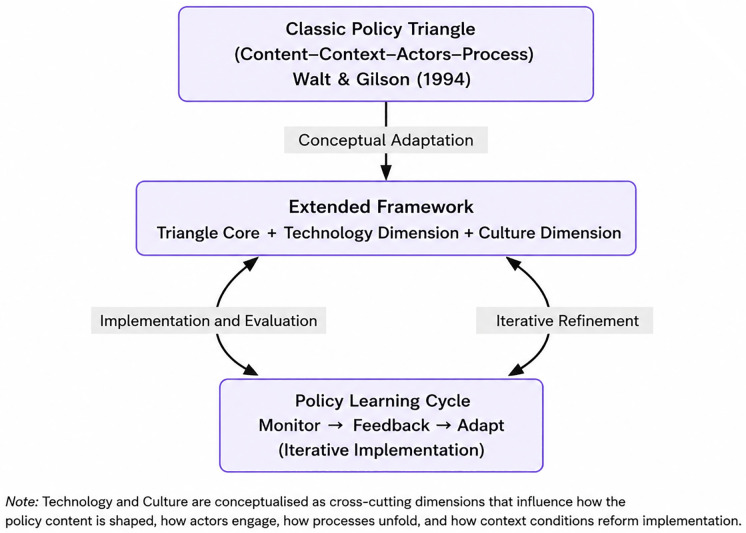
From classic policy triangle to extended digital–cultural framework and policy learning.

Fig 3 is included as a schematic synthesis to illustrate the evolution of the analytical framework; it is not required for application of the model but supports interpretation of policy learning dynamics.

The diagram shows progression from Walt and Gilson’s original policy triangle (content, context, actors, and process) to the extended framework (adding Technology and Culture as cross-cutting dimensions), followed by a policy learning cycle in which monitoring and feedback inform iterative adaptation and refinement of implementation over time.

The contribution of this paper is not a replacement of the classic framework, but a pragmatic and analytically grounded extension that improves its explanatory adequacy for large-scale transformation agendas, particularly where digital infrastructures and culturally patterned norms of decision-making, authority, and engagement are central to reform enactment.

### Key conclusions

Three overarching conclusions emerge from this analysis. First, extending Walt and Gilson’s Health Policy Analysis Triangle by making Technology and Culture explicit as cross-cutting dimensions strengthens its analytical adequacy for digitally mediated reforms without sacrificing the clarity and usability of the original framework. Second, the extended model demonstrates that variation in implementation outcomes can be more convincingly explained when digital infrastructure and sociocultural norms are treated as cross-cutting dimensions shaping actor agency, governance mechanisms, and policy processes, rather than as background context. Third, by bridging health system reform analysis with digital transformation scholarship, the framework offers a pragmatic and transferable policy dimension for examining reform implementation and learning in Saudi Arabia and other low- and middle-income health systems undergoing rapid transformation.

### Implications for research

For health policy and systems researchers, the extended model provides a structured way to analyze implementation variation that is often attributed vaguely to “context.” In digitally mediated reforms, researchers can operationalize the Technology dimension through constructs such as digital maturity, interoperability, workflow embedding, data governance, and capability development, drawing on global digital health strategy priorities. In culturally embedded reforms, the Culture dimension can be operationalized through constructs such as family-mediated decision-making, legitimacy and trust dynamics, gendered interaction expectations, and professional hierarchy. Methodologically, the framework supports comparative and mixed-methods designs by guiding analysts to trace how technology and culture interact with actor agency and implementation processes over time, and by encouraging explicit attention to feedback mechanisms and emergent adaptation rather than assuming linear implementation trajectories.

### Implications for teaching and capacity-building

For teaching health policy analysis, the extended model offers a clear pedagogical bridge between classic policy frameworks and contemporary realities. It can be used to help students and practitioners distinguish between (i) policy design intentions, (ii) organizational conditions for implementation, and (iii) the mediating role of digital systems and culturally patterned practices. As digital transformation becomes embedded in routine service delivery, integrating Technology and Culture into policy analysis curricula can strengthen analytical literacy and improve the practical relevance of training for Gulf and other low- and middle-income settings.

### Implications for policy formulation and implementation

For policymakers and reform leaders, the model underscores that successful reform requires alignment between policy intent and the infrastructures, digital and social, through which policy is operationalized. In practice, this implies prioritizing interoperability and usable information flows, investing in workforce capability and workflow integration, and designing accountability regimes that support learning rather than producing narrow compliance. It also implies treating culturally aligned engagement as a core implementation strategy, especially for patient-centred care and chronic disease management, where legitimacy, communication, and family participation shape adherence and outcomes. The framework encourages reform leaders to monitor unintended consequences of technology-enabled governance (for example, documentation burdens or metric-driven behavior) while strengthening culturally appropriate mechanisms for participation and trust.

### Limitations

This study has several limitations. First, the analysis is based on a secondary interpretation of qualitative data collected as part of a doctoral evaluation of the Saudi Health Sector Transformation Program. While this dataset provided valuable insights into reform implementation, the interview sample consisted of 53 participants from clustered and non-clustered hospital settings and should not be interpreted as representative of the Saudi health system as a whole. The findings should therefore be understood as indicative of patterns within the sampled settings rather than as evidence of system-wide reform outcomes. Second, the paper develops a conceptual extension of an existing policy framework rather than conducting a new empirical study, and therefore the analytical arguments rely on illustrative examples rather than exhaustive empirical testing. Finally, the findings are grounded in the Saudi reform context, which may limit direct generalization to other health systems without careful contextual adaptation.

## Conclusion

In summary, the extended policy triangle offers a locally contextualized but analytically applicable framework when adapted to context for SHSTP–Vision 2030 and comparable transformation agendas. By making Technology and Culture explicit, the framework better captures how contemporary reforms are enacted through digital infrastructures and culturally mediated interactions, thereby strengthening analytical clarity in implementation analysis and supporting more actionable learning for research and policy.

## Supporting information

S1 File Supplementary material: Expanded illustrative evidence supporting the extended policy triangle. This file provides additional illustrative qualitative excerpts supporting the vignettes presented in Section 5 of the manuscript, drawn from de-identified interviews conducted as part of the SHSTP evaluation.
(DOCX)
