# Supplementary material for: Applying an extended Health Policy Analysis framework to digitally mediated health system reform: Evidence from Saudi Arabia’s Health Sector Transformation Program
Source: PLoS One. 2026 Jun 8;21(6):e0350168. doi: 10.1371/journal.pone.0350168 (PMC13245744; doi:10.1371/journal.pone.0350168)
Supplement: S1 File — This file provides additional illustrative qualitative excerpts supporting the vignettes presented in Section 5 of the manuscript, drawn from de-identified interviews conducted as part of the SHSTP evaluation. (DOCX) [file pone.0350168.s001.docx]

**Supplementary Material S1**

**Expanded Illustrative Evidence Supporting the Extended Policy Triangle**

**Supplementary Material S1: Expanded Illustrative Vignettes**

This supplementary file provides additional illustrative qualitative excerpts that support the vignettes presented in Section 5 (Illustrative Vignettes) of the main manuscript. The excerpts are drawn from de-identified interviews conducted as part of the author’s doctoral evaluation of the Saudi Health Sector Transformation Program (SHSTP) (Alshehri, 2025). They are included to enhance transparency and to demonstrate how Technology and Culture operate as cross-cutting lenses shaping policy implementation, without introducing new analysis beyond that presented in the main text.

All quotations are anonymised, used illustratively, and approved under institutional ethics clearance.

**Vignette 1 (Expanded): Technology-enabled follow-up and continuity of care**

**Policy content:** Continuity of care and follow-up compliance under SHSTP
**Lens:** Technology
**Mechanism:** Workflow embedding and digital access
**Observed effect:** Follow-up perceived as easier and more continuous

Additional illustrative excerpts highlight how virtual tools altered follow-up routines in clustered hospitals:

“Before, patients missed appointments because of distance or transport. Now with online follow-ups, they are more likely to stay connected.”
*(physician)*

“It’s easier to check on patients after discharge because we can call or follow up online instead of waiting for the next visit.”
*(Physician)*

These accounts illustrate how digital follow-up tools reduced logistical barriers, reshaped service delivery routines, and supported continuity of care by embedding follow-up into everyday clinical workflows.

**Vignette 2 (Expanded): Interoperability and coordination across departments**

**Policy content:** Integrated care and coordination
**Lens:** Technology
**Mechanism:** Interoperability and data visibility
**Observed effect:** Variable coordination depending on system integration

Further excerpts demonstrate how uneven interoperability shaped coordination experiences:

“When all departments can see the same record, it saves time and avoids repeating tests.”
*(Patient)*

“Sometimes the system doesn’t update properly, so we still have to rely on phone calls or paper notes.”
*(Physician)*

These contrasting experiences illustrate how digital infrastructure readiness conditions the operational feasibility of integrated care, explaining variation in coordination outcomes despite shared policy intent.

**Vignette 3 (Expanded): Cultural norms and participation in decision-making**

**Policy content:** Patient-centred care and shared decision-making
**Lens:** Culture
**Mechanism:** Family-mediated authority and communication norms
**Observed effect:** Participation enacted as inclusion or substitution

Additional excerpts highlight how cultural norms shape engagement:

“Families expect to be involved, and often decisions are discussed with them first.”
*(Physician)*

“I don’t mind my family helping, but sometimes I feel the doctors don’t ask me directly.”
*(Patient)*

These accounts show how family involvement can both facilitate support and mediate patient voice, illustrating how culturally embedded norms shape the enactment of patient-centred policy objectives.

**Relationship with the Main Manuscript**

These expanded excerpts reinforce the analytical claims made in **Section 5** by demonstrating how Technology and Culture operate as structuring dimensions that shape actor roles, implementation processes, and the practical experience of reform. They do not introduce new findings but provide additional empirical grounding for the conceptual extension presented in the article.
